# Human Milk Oligosaccharide, Phospholipid, and Ganglioside Concentrations in Breast Milk from United Arab Emirates Mothers: Results from the MISC Cohort

**DOI:** 10.3390/nu11102400

**Published:** 2019-10-08

**Authors:** Paul McJarrow, Hadia Radwan, Lin Ma, Alastair K.H. MacGibbon, Mona Hashim, Hayder Hasan, Reyad Shaker Obaid, Farah Naja, Hamid Jan Jan Mohamed, Hessa Al Ghazal, Bertram Y. Fong

**Affiliations:** 1 Fonterra Research and Development Centre, Dairy Farm Road, Private Bag 11029, Palmerston North 4442, New Zealand; kevin.ma@fonterra.com (L.M.); bertram.fong@fonterra.com (B.Y.F.); 2 Department of Clinical Nutrition and Dietetics, College of Health Sciences, Research Institute of Medical and Health Sciences (RIMHS), University of Sharjah, Sharjah 27272, UAE; hradwan@sharjah.ac.ae (H.R.); mhashim@sharjah.ac.ae (M.H.); haidarah@sharjah.ac.ae (H.H.); robaid@sharjah.ac.ae (R.S.O.); 3 School of Health Sciences, Universiti Sains Malaysia, Kubang Kerian 16150, Malaysia; hamidjan@usm.my; 4 Department of Nutrition and Food Sciences, American University of Beirut, Beirut 1107 2020, Lebanon; fn14@aub.edu.lb; 5 Family Health Promotion Centre, Sharjah 27272, UAE; Hessa.ALGhazal@scf.shj.ae

**Keywords:** human milk, human milk oligosaccharides, phospholipids, sphingomyelin, gangliosides, LC–MS

## Abstract

Human milk oligosaccharides (HMOs), phospholipids (PLs), and gangliosides (GAs) are components of human breast milk that play important roles in the development of the rapidly growing infant. The differences in these components in human milk from the United Arab Emirates (UAE) were studied in a cross-sectional trial. High-performance liquid chromatography‒mass spectrometry was used to determine HMO, PL, and GA concentrations in transitional (5–15 days) and mature (at 6 months post-partum) breast milk of mothers of the United Arab Emirates (UAE). The results showed that the average HMO (12 species), PL (7 species), and GA (2 species) concentrations quantified in the UAE mothers’ transitional milk samples were (in mg/L) 8204 ± 2389, 269 ± 89, and 21.18 ± 11.46, respectively, while in mature milk, the respective concentrations were (in mg/L) 3905 ± 1466, 220 ± 85, and 20.18 ± 9.75. The individual HMO concentrations measured in this study were all significantly higher in transitional milk than in mature milk, except for 3 fucosyllactose, which was higher in mature milk. In this study, secretor and non-secretor phenotype mothers showed no significant difference in the total HMO concentration. For the PL and GA components, changes in the individual PL and GA species distribution was observed between transitional milk and mature milk. However, the changes were within the ranges found in human milk from other regions.

## 1. Introduction

Human milk (HM) is the complete food source for infants, providing all the nutrients required for growth in the early stages of life. There has been long interest in the composition of human milk and its changes with lactation and differences between mothers and between populations in different geographical locations or with different diets. Changes in the macronutrient composition of HM with respect to fat, protein, and lactose in different population cohorts have been investigated for many years [1].

As a group, human milk oligosaccharides (HMOs) are a major component of HM, forming the third most dominant component (12–18.6 g/L) after lactose (55–70 g/L) and fat 20–60 g/L) [2,3], and on average having a higher concentration than protein. However, whether they are a macronutrient in sensu stricto is open to interpretation [4]. Phospholipids (PLs) and gangliosides (GAs), which are constituents of the milkfat globule membrane (MFGM) that surrounds the lipid droplet, are minor components. Increasing evidence indicates that HMOs, PLs, and GAs play critical roles in the development of the growing infants and have been of increasing interest in recent years as infant formula manufacturers look to humanise their formulations.

HMOs are shown to play significant biological roles as prebiotics, antimicrobials, and immune modulators [5,6,7,8,9,10,11,12,13,14,15,16] for the growing infant. To date, there are over 200 oligosaccharides [17] reported in HM. One impact on the HMO profile from an individual mother is the absence (non-secretor) or presence (secretor) of a fully functional α-(1,2)-fucosyltransferase 2, which is coded by the FUT2 gene; milk produced by non-secretor mothers contains low concentrations of 2’-fucosyllactose (2’FL) as well as lacto-N-fucopentaose I (LNFP I) [5,18].

In addition to their essential roles in cell membrane structural integrity, PLs play critical roles in lung and brain development in the growing infant [19,20,21,22,23]. The PLs in HM are mainly found in the tri-layer of the MFGM and consist of sphingomyelins (SMs) and the glycerophospholipids phosphatidyl choline (PC) and its lyso species (L-PC), phosphatidyl ethanolamine (PE) and its lyso species (L-PE), phosphatidyl inositol (PI), and phosphatidyl serine (PS).

GAs are also important in neurological development, memory formation, and synaptic signal transduction, and are implicated in regulating the immune system and supporting gut maturation in the new born [24,25,26].

There are recent studies that report the changes in HMOs [27,28,29,30], PLs [31,32,33], and GAs [32,33,34,35] across lactation for various population cohorts; however, to our knowledge, there is no specific study reported for the UAE or other Middle Eastern populations. In this study, the HMO, PL, and GA concentrations in transitional (5–15 days) and mature milk (at 6 months post-partum) were measured in a cross section of UAE mothers’ breast milk samples, collected from a wider mother–infant study cohort (MISC) [36]. The information obtained from this study allows comparison with other similar studies on HMO, PL, and GA levels in HM from other population cohorts to better address the hypothesis that geography and ethnicity impact the levels of these HM bioactives [28].

## 2. Materials and Methods

### 2.1. Study Setting and Population

A randomly selected subsample of breast milk samples (transitional milk period, days 5 to 15 post-partum, *n* = 41, and mature milk at 6 months post-partum, *n* = 40) were made from a large cohort collected as part of the MISC in the UAE to comprehensively investigate maternal and infant factors in relation to child health outcomes, as well as early-life determinants of non-communicable diseases, through integration of sociodemographic, dietary, lifestyle, anthropometric, and biological and cognitive data [36].

Arab participants were recruited from antenatal clinics in three main public governmental hospitals, and seven primary health care clinics and mother and child centres in the Emirates of Sharjah, Dubai, and Ajman. The inclusion criteria were pregnant women who were Emirati or Arab expatriate; aged 19–40 years; singleton pregnancy; within the third trimester of pregnancy (27–42 weeks of gestation); free of chronic diseases (including diabetes, hypertension, kidney disease, cancer, and others), autoimmune disorders, infections with the human immunodeficiency virus, or hepatitis in preconception; receiving antenatal care in any of the above-mentioned clinics; and expected to give birth in a participating public hospital and remaining in the UAE during the timeline of the study. The exclusion criteria were as follows: multiple pregnancy, high risk pregnancy or pre-eclampsia, and history of chronic diseases [36].

The study was approved by the Research and Ethics Committee, University of Sharjah (REC/14/01/1505) and by Al Qassimi Clinical Research Centre Ethical Research Committee (REC Reference Number: 215 12015-03), by the Ministry of Health Ethical Research Committee (R02), and by Dubai Health Authority (DSREC-0/2016).

### 2.2. Phospholipid, Ganglioside, and Oligosaccharide Analysis

Extraction of HM samples and analysis using HPLC-MS was as described previously for PLs [31] and GAs [34]. Briefly, 0.25 mL of HM samples was extracted using the modified Svennerholm and Fredman [37] method described by Fong et al. [38]. The non-polar (lower) phase was diluted to 5 mL with choroform/methanol (1:2) and an aliquot was used for quantification of PC, PS, PI, PE, SM, L-PC, and L-PE. Separation and quantification were as described previously [38] with individual PL species separated using an ACQUITY UPLC system (Waters, Milford, MA, USA) equipped with an APS-2 Hypersil column (150 mm × 2.1 mm, 3 Um, Thermo Electron Corporation, Waltham, MA, USA). The eluate from the HPLC was directed into a TSQ Quantum mass spectrometry (Thermo Electron Corporation, Waltham, MA, USA) with individual PL species detected either by precursor ion or neutral loss experiments [38]. The polar (upper) phase from the extraction was diluted to 10 mL with methanol/water (1:1), and then GA classes (disialoganglioisde 3 (GD3) and monosialoganglioside 3 (GM3)) were separated on an Agilent 1100 series HPLC system (Santa Clara, CA, USA) equipped with an APS-2 Hypersil column (150 mm × 2.1 mm, 3 mm), interfaced to a SCIEX 6500 QTrap mass spectrometer (AB SCIEX, Framingham, MA, USA) and quantified as described by Ma et al. [26] using multiple reaction monitoring in negative mode.

HMOs (3’-sialyllactose (3’SL), 6’-sialyllactose (6’SL), 6’-sialyllactosamine (6’SLN), disialyllactose (DSL), 3’-sialyl-3-fucosyllactose (3’S3FL), LS-tetrasaccharide a/b (LSTa/b), LS-tetrasaccharide (LSTc), lacto-N-neotetraose (LNnT), lacto-N-tetraose (LNT), Lacto-N-fucopentaose (LNFP total against LNFP I as standard), 2’-fucosyllactose (2’FL), and 3-fucosyllactose (3FL)); were analysed for, and in all cases, the sialic acid is N-acetyl neuraminic acid. HMOs were separated using a Luna hydrophilic interaction liquid chromatography column (150 mm × 2.1 mm, 3 um, Phenomenex, Torrence, CA, USA) on an Agilent 1100 series HPLC [30]. The HPLC system was coupled to a SCIEX 6500 QTrap mass spectrometer, operated in negative ion mode, and HMOs were quantified as described [30] using 3’-sialyllactosamine as an internal standard.

### 2.3. Statistical Analysis

Statistical analysis was conducted using one-way analysis of variance (ANOVA), where difference among the means is significant when *p* < 0.05 (MiniTab Release 17.2.1 2016, MiniTab Inc., State College, PA, USA).

## 3. Results

### 3.1. HMO

Seven acidic (3’SL, 6’SL, 6’SLN, 3’S3FL, DSL, LSTa/b, and LSTc) and five neutral (LNnT, LNT, LNFP (total), 2’FL, and 3FL) oligosaccharides were measured.

The average HMO concentration in transitional milk (8204 ± 2389 mg/L; Table 1) was significantly higher than that measured in mature milk (3905 ± 1466 mg/L; Table 1). This trend was also reflected at the individual HMO level (Table 1). When secretor and non-secretor milk samples were considered separately (based on a non-secretor milk having a 2’FL concentration <50 mg/L), the total HMO concentrations for both groups were not significantly different for either transitional or mature milk (Table 1).

The proportion of the acidic HMOs in transitional milk (18%) was higher than that measured in mature milk (7%); concomitantly, the neutral HMOs in transitional milk were 82% of total HMOs measured, compared with 93% in mature milk. When considered separately, secretors and non-secretors were not significantly different in this aspect. The HMO species for which there was a significant difference in concentration between secretor and non-secretor milk samples were 2’FL, 3FL, and 3’S3FL (Table 1). The secretor milk samples were higher in 2’FL and lower in 3FL and 3’S3FL compared with non-secretor milk samples.

### 3.2. Phospholipids

The average total PL concentration (± SD) measured in the transitional milk (269.0 ± 89.2 mg/L) was significantly (*p* < 0.05) higher than that measured for mature milk (219.6 ± 85.0 mg/L, Table 2). The concentration of PE, SM, and L-PE did not change significantly (*p* > 0.05) between transitional and mature milk, but PI, PC, PS, and L-PC decreased significantly in concentration in mature milk (Table 2).

The relative distribution of the five different PL classes (PI, PC, PE, PS, and SM) and L-PC and L-PE is presented in Table 2. Because of the changes in individual PL species concentration, significant changes in the relative distribution of PL classes were observed, with PE increasing from 25% in transitional milk to 36% in mature milk (6 months post-partum), while PC and PS decreased from 25% to 14% and 11% to 7%, respectively. SM remained the dominant PL class during both lactation milk time points, and only increased slightly (Table 1). Little change in the PI distribution was observed over the two-time points, decreasing from 4% to 3%.

### 3.3. Gangliosides

The average total GA (TGA) concentration measured in the UAE mothers’ transitional milk (21.2 ± 11.46 mg/L) and mature milk (20.2 ± 9.8 mg/L) was not significantly different (*p* > 0.05) across the two-time points. However, the relative distribution of ganglioside classes, GD3 and GM3, changed across the two-time points from 56% and 44%, respectively, for transition milk, to 9% and 91% respectively, for mature milk (Table 2), with both GM3 and GD3 showing significant changes in concentration.

## 4. Discussion

HM is considered the “gold standard” for an infant’s nutrition, to which infant formula manufacturers strive to emulate in both nutrient composition and performance. HM composition varies considerably between individual mothers and over lactation, and so could be considered to be personalised to each infant that is breastfed. Various factors such as diet, geography, ethnicity, milk collection time, and genetics have been implicated to have a significant influence on the HMO, PL, and GA composition in HM, but most of the data obtained to date indicate that the stage of lactation is perhaps the primary factor that has the greatest influence on HM composition [27,28,29,39]. There are several recent studies reporting the composition of HM, trying to gain a better understanding of the changes in the HMOs and complex lipids (PLs and GAs) through lactation of different geographical population cohorts [28,31,32,34,35,39,40]. However, this is the first study that looks at the transitional and mature milk from UAE mothers, helping to address geographical variation in HMOs, PLs, and GAs.

### 4.1. Human Milk Oligosaccharides

**Figure 1:**
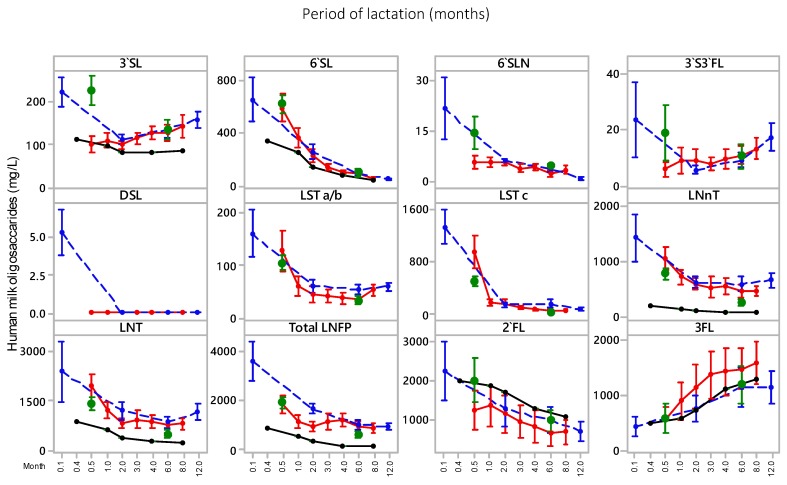
Human milk oligosaccharide (HMO) concentrations in the United Arab Emirates (UAE) mothers’ milk (●) across the transitional milk and mature milk periods, plotted against the lactational trend reported by Ma et al. [30] for a Chinese cohort (●) and Malaysian cohort (●), and by Austin et al. [29] for a Chinese cohort (●). 3’SL, 3’-sialyllactose; 6’SL, 6’-sialyllactose; 6’SLN, 6’-sialyllactosamine; DSL, disialyllactose; 3’S3FL, 3’-sialyl-3-fucosyllactose; LSTa/b, LS-tetrasaccharide a/b; LSTc, LS-tetrasaccharide; LNnT, lacto-N-neotetraose; LNT, lacto-N-tetraose LNFP, lacto-N-fucopentaose I; 2’FL, 2’-fucosyllactose; 3FL, 3-fucosyllactose.

The UAE mothers’ transitional breast milk samples had significantly higher average total HMO concentrations (8204 ± 2389 mg/L) compared with the mature milk samples (3876 ± 1403 mg/L). The largest decrease in the HMOs over these two lactation timepoints was observed with the acidic oligosaccharide, LSTc, which decreased by 98% from 488 ± 224 mg/mL in transitional milk to 11 mg/L in the six months post-partum mature milk (Table 1). The only HMO to increase across this period was 3FL (Table 1). This trend is in line with lactational trend data reported in the literature [27,29,30] (Figure 1). While only five neutral oligosaccharides (2’FL, 3FL, LNT, LNnT, and total LNFP) were measured in this study, they made up a significant proportion (82% and 93% for transitional and mature milk, respectively) of the total HMOs measured in this study, with the acidic HMOs making up 18% and 7% for transitional and mature milk, respectively. This finding is consistent with those reported by Ma et al. [30] for their Malaysian and Chinese cohort of 89%–91% and 8.5%–11% of neutral and acidic HMOs, respectively, and other similar studies [27,28,29], despite differences in the range of HMOs and respective concentrations being different.

The individual HMO levels measured in this study for the UAE breast milk samples were also in a range similar to that reported by Ma et al. [30] for Chinese and Malaysian mothers (Table 3 and Figure 1); and Larsson et al. [41], Coppa et al. [42], Bao et al. [43], and Austin et al. [29] for the common HMOs measured for the corresponding time points, except for 3’SL, LNT, and LNnT where Austin et al. [29] reported lower HMO lactational results (Table 3 and Figure 1).

HMOs have been implicated not just to provide anti-infective protection for the infant, but also as being involved as immune modulators, and may play a key role in gut maturation of the rapidly growing infant. Higher HMO concentrations in colostrum and transitional milk may be the consequence of increased protection required for the vulnerable infant during the early few days of life. The changes in HMO levels over the course of lactation [29,30] may reflect changes in the development stages of the growing infant, and a requirement for specific compositions of these HMOs.

On the basis of the 2’FL concentrations, 26% of the UAE mothers in this cohort expressed a non-secretor phenotype, having a 2’FL concentration <50 mg/L in their breast milk samples [29,44]. 2’FL (and LNFP I) are products of α-(1,2)-fucosyltransferase 2, which is coded by the FUT2 gene that is supposedly non-functional in non-secretor mothers. However, in the study of Austin et al. [20], 2’FL was found to be not completely absent in the non-secretor mothers, as was the case in this study. The secretor/non-secretor frequency is known to vary with geography and racial difference, with 22.5% non-secretor phenotype reported for the Han Chinese population, Eastern China region [29]; 37% reported for the Chinese cohort (Guangzhou); and 17% reported for Malay mothers [30]. The typical frequency of non-secretor phenotype reported by Azad et al. [39] was 28% for Caucasian mothers, while Asian mothers had higher non-secretor frequency at 40%; however, the Asian mothers’ sub-ethnic groups were not defined. There is no current information as to the non-secretor frequency for the UAE population. Azad et al. [30] also reported that the non-secretor group had significantly less HMOs than the secretor group. For the HMOs measured in this study, however, there was no significant difference (*p* > 0.05) in the average total HMOs between secretor and non-secretor mothers for either their transitional milk (8292 ± 2516 mg/L versus 6994 ± 1905 mg/L, respectively) or their mature milk (4289 ± 1791 mg/L versus 4317 ± 1857 mg/L, respectively) (Table 1).

One important consideration in assessing crude population figures for HMO species is the impact that the percentage of non-secretors in each study population has on the average or mean 2’FL and 3FL concentrations that would be reported for the full cohort; the higher the percentage of non-secretors in a population, the lower the average 2’FL and higher the average 3FL concentrations for the cohort as a whole. Furthermore, it is not only the crude 2’FL and 3FL figures that are impacted, but also the figures for other oligosaccharides such as LNFP I and 3’S3FL. For example, in this study, the mature milk results for the full cohort showed that 2’FL and 3FL concentrations are relatively similar, which was also shown by Ma et al. [30] and Austin et al. [29], but if the non-secretor percentage was lower, then 3FL would be lower than 2’FL at later points in lactation, as evidenced in the limited data of Larsson et al. [41], which is the analysis of secretors alone, and the results of this study. Figure 1 also emphasises the impact of lactation on interpreting which oligosaccharides are in highest concentration, because, at six months in all four cohorts (two Chinese, one Malaysian, and one UAE), the concentrations of 3FL and 2’FL are very similar.

### 4.2. Phospholipids

The average total PL concentrations observed with HM samples of UAE mothers were within the typical ranges reported for human breast milk for other geographical population cohorts (Table 4). In this study, the UAE transitional milk had an average total PL concentration that was significantly higher (*p* < 0.05) than that measured for the six-month mature milk. While this trend is consistent with the majority of the published data [31,33,45,46,47,48], the trend reported from some studies showed the total PL concentration in colostrum and transitional milk was either much lower than [49,50,51] or the same as [52] that in mature milk (Table 4).

**Figure 2:**
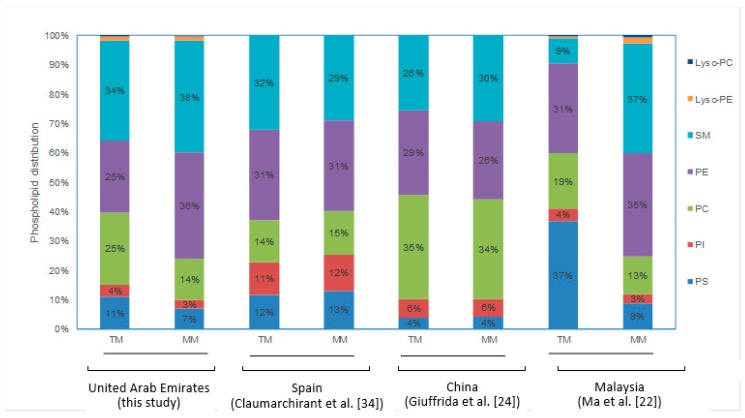
Distribution of phospholipid classes in transitional milk (TM) and mature milk (MM; six months post-partum) in UAE mothers’ milk, and from other geographic cohorts. SM, sphingomyelins; PC, phosphatidyl choline (PC); L-PC, PC lyso species; PE, phosphatidyl ethanolamine; L-PE, PE lyso species; PI, phosphatidyl inositol; PS, phosphatidyl serine.

At the individual PL class level, changes in the relative distribution of the individual PL classes were observed over the transitional milk and mature milk periods (Figure 1). There was a significant increase in the relative amount of PE from 25% in transitional milk to 36% in the UAE mature milk samples, while PC and PS both decreased, the former from 25% to 14% and the latter from 11% to 7%. SM increased only slightly, while PI decreased slightly. Similarly, changes in the relative distribution between transition milk and mature milk was observed for a Malaysian HM cohort [31] (Figure 2). In contrast, however, the PL distribution was relatively constant for Spanish [34] and Chinese [24] breast milk cohorts (Figure 2).

However, across the mature milk period, three recent studies [31,32,46] and two earlier studies [49,52] showed that the individual PL class distribution remained relatively constant through the mature milk period, despite changes in their absolute concentrations.

Variation in absolute PL concentrations may be attributable to a variety of factors, such as time of sampling protocols (full breast expression, time of sampling, and breast variation [53]), diet, geographic, and even metabolic stage and gestational age at birth, in addition to different analytical methods used [54,55,56]. However, the fact that the relative distribution of the individual PL class remains constant through the mature milk period indicates that some metabolic controls are maintained over the biosynthesis of these PL classes, perhaps to maintain the integrity of the MFGM structure.

Changes in the individual PL distribution observed between early milk and mature milk may be the consequence of the changing structure. It is reported that colostrum and transitional milk has much larger fat droplet size than that of mature milk [57]. In fact, Cohen et al. [58] reported the phospholipid composition of the mammary epithelial cell regulated the lipid droplet size, rather than the cellular triglyceride content; this phospholipid composition is critical in maintaining membrane structure integrity as the lipid droplet size changes.

### 4.3. Gangliosides

In HM, GAs are present predominantly as the GM3 and GD3 classes (which have the same polar head group, but different sphingosine and fatty acid). Typically, GD3 is present at relatively high concentrations in colostrum and transitional HM, making up approximately 30%–80% of the TGAs, but decreasing to 8%–25% by four to six months post-partum. Conversely, the relative proportion of GM3 is higher in mature milk [33,35,59,60,61].

However, there is no clear consistent lactational trend observed for TGA concentration. While a few studies show colostrum and transitional milk to contain the highest TGA concentration [34,59], other studies show the opposite [60,62]. Similarly, the TGA lactation trend over the mature milk period is also not clear. While the studies of Thakkar et al. [32] and Ma et al. [34] showed a gradual increase in the TGA level over the mature milk period (of four months and eight months duration, respectively), from the data published for the Malaysian cohort [34], this gradual increase did not appear to be sustained: the average TGA levels dropped from ~25.3 ± 15.7 mg/L at 6 months to 16.6 ± 8.5 mg/L at around 12 months post-partum.

In this study, there was no significant difference between the average TGA results measured between the transitional milk (21.18 ± 11.46) and the mature milk period (20.18 ± 9.75), with GD3 making up 55% of the TGAs in transition milk, decreasing to 8% at six months post-partum. The change in the GA class distribution is consistent across other published cohort studies [34,35] (Table 5), suggesting that the biosynthesis of GAs is under metabolic control, and different classes may be required for different stages of the development and growth for the breast fed infant. Interestingly, Thakkar et al. [32] reported that HM from mothers with male infants had higher energy content and lipids compared with that mothers of female infants; these differences may be because of differences in nutritional requirements to support specific growth and development patterns between the two sexes [23]. However, in the current study, there were no significant differences in the GA concentrations of either the transitional milk or mature milk breast milk from mothers who had male or female infants. The average TGA levels observed with the UAE cohort were similar to those reported for the Malaysian and Chinese cohorts (25.3 ± 15.7 mg/L and 22.9 ± 9.9 mg/L, respectively [34,35]), but higher than those reported by Thakkar et al. [32] for their Singapore cohort (4.6–5.6 mg/L) and Giuffrida et al. [33] for their Chinese cohort (11.0 ± 5.0 mg/L).

## 5. Conclusions

This study provides new information about the HMO, PL, and GA concentrations in breast milk specific to UAE mothers. Despite reports indicating that human milk composition varies across different geographies and ethnicities, the average HMO, PL, and GA concentrations measured in the MISC study from UAE were within the typical ranges reported for other ethnic cohorts, especially data obtained by the same methodologies. There appear to be more similarities than differences for HMOs, PLs, and GAs in HM from different geographical locations and ethnicities. The similarity in the range of concentrations of HMO, PL, and GA between different cohorts suggests they each have specific biological and functional roles linked to timing of lactation.

## Figures and Tables

**Table 1:** Human milk oligosaccharide (HMO) concentration in transitional and mature human milk from United Arab Emirates (UAE) mothers. 3’SL, 3’-sialyllactose; 6’SL, 6’-sialyllactose; 6’SLN, 6’-sialyllactosamine; DSL, disialyllactose; 3’S3FL, 3’-sialyl-3-fucosyllactose; LSTa/b, LS-tetrasaccharide a/b; LSTc, LS-tetrasaccharide; LNnT, lacto-N-neotetraose; LNT, lacto-N-tetraose LNFP, lacto-N-fucopentaose I; 2’FL, 2’-fucosyllactose; 3FL, 3-fucosyllactose.

Sample	3’SL	6’SL	6’SLN	DSL	3’S3FL	LSTa/b	LSTc	2’FL	3FL	LNnT	LNT	LNFP	Total HMO
Transitional (all) (*n* = 41)	226 ± 107 ^a^	621 ± 212 ^a^	15 ± 15 ^a^	2.2 ± 2.3 ^a^	19 ± 21 ^a^	104 ± 46 ^a^	488 ± 224 ^a^	2021 ± 1776 ^a^	581 ± 868 ^a^	765 ± 350 ^a^	1429 ± 693 ^a^	1932 ± 762 ^a^	8204 ± 2388 ^a^
Non-secretor	256 ± 144	562 ± 232	20 ± 23	1.8 ± 1.9	48 ± 49	124 ± 52	456 ± 243	4.3 ± 8.6	1599 ± 119	990 ± 524	1917 ± 973	1490 ± 528	7466 ± 1812
Secretor	216 ± 91	643 ± 204	13 ± 11	2.4 ± 2.4	8.1 ± 7.0	98 ± 42	500 ± 220	2761 ± 1497 ^#^	208 ± 112 ^#^	682 ± 220	1250 ± 460	2094 ± 777 ^#^	8475 ± 2541
Mature (all) (*n* = 40)	134 ± 69 ^b^	91 ± 108 ^b^	5 ± 1 ^b^	0.2 ± 0.4 ^b^	10 ± 14 ^a^	31 ± 25 ^b^	11 ± 8 ^b^	997 ± 885 ^a^	1194 ± 106 ^b^	250 ± 188 ^b^	504 ± 337 ^b^	650 ± 416 ^b^	3876 ± 1403 ^b^
Non-secretor	181 ± 98	81 ± 52	4.9 ± 2.2	0.1 ± 0.2	25 ± 20	28 ± 18	6.7 ± 5.3	4.0 ± 2.8	2526 ± 113	187 ± 113	420 ± 276	548 ± 300	4009 ± 1104
Secretor	116 ± 44	95 ± 124	4.4 ± 0.6	0.3 ± 0.5	4.6 ± 2.4 ^#^	32 ± 28	13 ± 8 ^#^	1374 ± 746 ^#^	688 ± 398 ^#^	273 ± 207	536 ± 357	689 ± 451	3826 ± 1515

Values (in mg/L) are means ± standard deviation. Different symbol pairings signify statistically significant values (*p* < 0.05); when comparing transitional and mature milk individual HMO and total, differences in groups are represented by ^a,b^. ^#^ is used if the difference between secretor/non-secretor groups is significant.

**Table 2:** Phospholipid (PL) and ganglioside (GA) concentrations in transitional and mature breast milk. SM, sphingomyelins; PC, phosphatidyl choline (PC); L-PC, PC lyso species; PE, phosphatidyl ethanolamine; L-PE, PE lyso species; PI, phosphatidyl inositol; PS, phosphatidyl serine; GD3, disialoganglioisde 3; GM3, monosialoganglioside 3.

Time Point	Phospholipids								Gangliosides		
	PI	PE	PC	SM	PS	L-PE	L-PC	Total PL	GM3	GD3	Total GA
Transitional (*n* = 41)	11.2 ± 5.5 ^a^ (4%)	66.3 ± 27.16 ^a^ (25%)	66.4 ± 32.87 ^a^ (25%)	91.2 ± 26.38 ^a^ (34%)	28.5 ± 13.29 ^a^ (7%)	3.7 ± 2.37 ^a^ (1.4%)	1.7 ± 0.98 ^a^ (0.6%)	269.0 ± 89.2 ^a^	9.47 ± 8.37 ^a^ (45%)	11.71 ± 9.46 ^a^ (55%)	21.18 ± 11.46 ^a^
Mature (*n* = 40)	6.5 ± 3.61 ^b^ (3%)	80.0 ± 35.35 ^a^ (36%) ^#^	30.2 ± 22.07 ^b^ (14%) ^#^	82.9 ± 29.21 ^a^ (38%)	16.1 ± 6.99 ^b^ (7%)	3.1 ± 1.99 ^a^ (1.4%)	0.9 ± 0.63 ^b^ (0.4%)	219.6 ± 85.0 ^b^	18.62 ± 9.69 ^b^ (92%) ^#^	1.57 ± 2.24 ^b^ (8%) ^#^	20.18 ± 9.75 ^a^

Values (in mg/L) are means ± standard deviation; the relative distribution (%) of the individual phospholipid classes and ganglioside classes are in parenthesis. In a column, values with the same superscript indicate no significant difference (*p* > 0.05) between the transitional and mature milk samples; when comparing transitional and mature milk individual phospholipids and gangliosides and total, differences in concentration are represented by ^a,b^. ^#^ is used for when the differences in the relative percentages of the individual phospholipids and gangliosides is significant between the transitional and mature milk.

**Table 3:** Human milk oligosaccharide mean concentrations reported in the literature for both transitional and mature milk about six months *post partum* (mg/L, except for Austin et al. [20], which is mg/kg).

Reference	Milk (Post Partum)	n	3’SL	6’SL	6’SLN	DSL	3’S3FL	LSTa/b	LSTc	2’FL	3FL	LNnT	LNT	Total LNFP
Larsson et al. [41] ^a,b^ Denmark	Mature (5 month)	15	492	156				149	33	2989	229	578	703	2692
Coppa et al. [42] Italy	Transition (4 days)	18								3930	340	2040	840	1650
Bao et al. [43] USA	Transition (9–21 days)		76	396				74 ^c^	148					
Austin et al. [20] (Table 4) China	Transition (5–11 days)	90	110	340						2600	510	180	880	1157
	Mature (4–8 months)	90	83	45						1300	1300	59	250	199
Ma et al. [21] China	Transition (14 days)	20	100	593	6	3	6	127	941	1281	543	1033	1979	1870
	Mature (6 months)	20	127	83	2	0	11	33	47	704	1476	446	785	945
Ma et al. [21] Malaysia	Mature (6 months)	21	135	84	4	0	9	84	145	1003	1146	571	867	1036
This study UAE	Transition (6–14 days)	41	226	621	15	2	19	104	488	2021	581	765	1429	1932
This study UAE	Mature (6 months)	40	134	91	5	0	10	31	11	997	1194	250	504	650

^a^ secretors only. ^b^ calculated from nmol/mL data. ^c^ LSTb alone.

**Table 4:** Human milk phospholipid concentration reported in the literature for both transitional and mature milk (modified from Ma et al. [31]) ^a^.

Total PL Ranges (mg/L)		Country	Reference
Transitional Milk	Mature Milk		
390 ± 50‒440 ± 73 (8‒15, 20)	370 ± 106‒405 ± 80 (22–36, 40)	Germany	Harzer et al. [52]
310 ± 30 (11, 5)	270 ± 30 (23, 5 pooled)	USA	Bitman et al. [47]
158 (11, 17)	114 (23, 19)	Spain	Sala-Vila et al. [48]
148 (7, 6)	133–227 (20–84, 6)	USA	Bitman et al. [49]
973 (14, 10)	1023‒1298 (42‒112, 10)	USA	Clark et al. [50]
185 (6‒15, 45)	182 (>16, 45)	Denmark	Zou et al. [51]
550 ± 260 (6–10, 7)	450 ± 260 (30, 16)	France	Gracia et al. [45]
	230 ± 49–242 ± 82 (30–120, 50)	Singapore	Thakkar et al. [32]
437 ± 23–535 ± 26 (6–15, 44)	260 ± 3–422 ± 13 (16–360, 44)	Spain	Claumarchirant et al. [46]
266 ± 57 (6‒14, 12)	170 ± 80‒219 ± 92 (60–365, 132)	Malaysia	Ma et al. [31]
285 ± 144 (6–15, 81)	242 ± 114 (16–240, 345)	China	Guiffrida et al. [33]
269 ± 89 (6–14, 41)	220 ± 85 (180, 40)	UAE	This study

^a^ Lactational period, in days, and number of samples are given in parentheses. Data from Sala-Vila et al. [48] recalculated from total phospholipid data provided using average molecular masses of 758.4, 864.4, 787.1, 737.6, and 754.4 for PC, PI, PS, PE, and SM, respectively; data from Bitman et al. [49] recalculated from graph using termed mothers only.

**Table 5:** Human milk mean GM3, GD3, and total ganglioside concentrations reported in the literature for both transitional and mature milk (mg/L ± standard deviation) ^a^.

Reference	Milk	*n*	GM3	GD3	Total GA
Giuffrida et al. [40] China	Colostrum/Transition (0–11 days)	450	3.8 ± 0.4 (47)	4.3 ± 0.9 (53)	8.1
Ma et al. [34] Malaysia	Transition	12	8.3 ± 4.8 (44)	10.6 ± 4.3 (56)	18.9 ± 6.6
This Study UAE	Transition (5–15 days)	41	9.5 ± 8.4 (45)	11.7 ± 9.5 (55)	21.2 ± 11.5
Ma et al. [34] Malaysia	Mature (6 months)	42	21.4 ± 13 (85)	4.3 ± 5.5 (15)	25.3 ± 15.7
Ma et al. [35] China	Mature (6 months)	20	21.4 ± 9.5 (93)	1.5 ± 1.4 (7)	22.9 ± 9.9
This Study UAE	Mature (6 Months)	40	18.6 ± 9.7 (92)	1.6 ± 2.2 (8)	20.2 ± 9.8

^a^ % of total GA is given in parenthesis.
